# Investigating students’ attitudes towards translation technology: The status quo and structural relations with translation mindsets and future work self

**DOI:** 10.3389/fpsyg.2023.1122612

**Published:** 2023-02-16

**Authors:** Sha Tian, Lingxiao Jia, Zhining Zhang

**Affiliations:** ^1^School of Foreign Languages, Central South University, Changsha, China; ^2^School of Foreign Languages and Cultures, Ningxia University, Yinchuan, China

**Keywords:** translation technology teaching, attitudes towards translation technology, translation mindsets, future work self, structural relations

## Abstract

**Introduction:**

Despite the growing attention paid to the research of translation technology teaching (TTT), there is still a lack of studies on students’ attitudes and the motivational factors in relation to it. To this end, the paper reports on a questionnaire-based study that describes students’ attitudes towards translation technology (in the Chinese MTI context) and explores its structural relations with translation mindsets and future work self.

**Methods:**

Data were collected from 108 grade 2021 MTI students of three selected Chinese universities and analyzed using descriptive statistics and structural equation modeling (SEM).

**Results:**

The results demonstrate that Chinese MTI students’ overall attitudes towards translation technology are slightly positive. So far, they perceive translation technology to be slightly effective for translation and are slightly mindful of it. They are slightly influenced by teachers and still feel inhibited when learning and using it. Furthermore, the results also indicate that growth translation mindsets positively influence students’ attitudes towards the effectiveness of translation technology, teacher influence, exhibition to translation technology, and mindfulness about translation technology, whereas fixed translation mindsets only negatively predict students’ teacher influence. Likewise, future work self-salience positively associates with students’ attitudes towards the effectiveness of translation technology and mindfulness about translation technology, while future work self-elaboration positively relates to students’ exhibition to translation technology. Among them, growth translation mindsets are the strongest predictor for all attitudes components.

**Discussion:**

Theoretical and pedagogical implications are also discussed.

## 1. Introduction

Translation technology denotes a variety of technologies employed by translators in the translation process, for instance, search engines, CAT tools, corpora use, machine translation, localization tools, quality assurance tools, resource management software, and so on (Man et al., 2020; He et al., 2022). As confirmed by numerous surveys (Biau-Gil and Pym, 2006; Bowker and Marshman, 2010; Krüger, 2018), translation technology has brought drastic changes to the translators’ workflow (Sikora, 2014; Yan and Wang, 2022) and is becoming increasingly entrenched in today’s translation profession. This “technological turn” (Cronin, 2010) has prompted the ratification of instrumental competence as the significant component of translation competence (EMT, 2017; PACTE, 2018). Accordingly, translation training programs worldwide have incorporated translation technology teaching (TTT) into their curricula. Nevertheless, it is not only necessary for translation educators to introduce the technological content but also devise effective pedagogy for a complete response to the technology-induced shift (Sánchez Ramos, 2022).

Therefore, TTT-related research is vital and has already become a well-delineated topic in translation studies. A review of the TTT literature has shown that translation scholars mainly follow three lines of inquiry. (1) Some scholars discussed the didactic necessities of TTT (Jiménez-Crespo, 2013; Kenny and Doherty, 2014; Vargas-Sierra, 2014; Massey and Ehrensberger-Dow, 2017; Tao and Wang, 2022) and its impact on the translation training programs (Bowker and Marshman, 2010; Pym, 2011; Rodríguez-Inés, 2013; Rico, 2017; Mellinger, 2018; Man et al., 2020). (2) Others also documented and reflected on its existing teaching approaches (Pym et al., 2006; Rodríguez-Castro, 2018; Rothwell and Svoboda, 2019; Zhang and Vieira, 2021). These studies have generally confirmed the indispensable role played by TTT in translation education and identified the growing TTT-industry gap, thus advocating for pedagogical modification. (3) In response, a substantial bulk of study has been done on TTT course design with detailed accounts of what to teach and how to teach. In terms of what to teach, scholars have conducted enormous research on its teaching contents and resources in various sub-topics, such as computer-assisted translation (Doherty and Moorkens, 2013; Enríquez Raído, 2013; Shuttleworth, 2017; Rodríguez-Castro, 2018), term management (Martínez and Benítez, 2009), localization (Jiménez-Crespo and Tercedor, 2011; Jiménez-Crespo, 2013), statistical machine translation (Doherty and Kenny, 2014), machine translation and post-editing (Moorkens, 2018; Guerberof Arenas and Moorkens, 2019), technical writing (Tao et al., 2020), specialized corpora (Rodríguez-Inés, 2013), python-based repository (Krüger, 2021), and data science (Yan and Wang, 2022). Meanwhile, topics like the technological skill-sets that student translators are required to acquire were often discussed (Austermühl, 2013; Gaspari et al., 2015; He and Tao, 2022). In terms of how to teach, scholars have delved into designing instructions underlying socio-constructive learning, for instance, simulations (González Davies and Enríquez Raído, 2016; Krüger, 2016; Killman, 2018; Mellinger, 2018), projects (Guerberof Arenas and Moorkens, 2019; Mitchell-Schuitevoerder, 2020; He and Tao, 2022), learning portfolio empowerment (Calvo, 2017; Rico, 2017), the ecosystem of translator workstation (Mo and Man, 2017), autonomous learning (Shuttleworth, 2017; Nunes Vieira et al., 2021), cross-module integration (Doherty and Kenny, 2014; Gaspari et al., 2015; González Pastor and Rico, 2021), and virtual and blended learning (Rodríguez-Castro, 2018; Wang and Wang, 2021; Sha et al., 2022).

In summary, translation scholars have generated valuable recommendations about teaching translation technology. However, the existing literature predominantly emphasizes the course design itself, painting an impersonal picture of students as the recipients. Little research has investigated the human factors associated with students’ translation technology learning and use, such as their attitudes (Liu et al., 2022). In fact, attitudes, which gain universal recognition as a critical factor in students’ acceptance of technologies (Vandewaetere and Desmet, 2009; Zarrinabadi et al., 2022), are very much related to the degree to which TTT is successful (Alcina et al., 2007). Positive attitudes can stimulate students’ perseverance towards technology and raise their behavioral intention to learn and use it, whereas negative attitudes would trigger psychological barriers that might impede these processes (Vandewaetere and Desmet, 2009; Zarrinabadi et al., 2022). Given its significance to TTT, it seems advisable to call for research on students’ attitudes. In particular, the attitudes’ antecedents, or rather the individual motivational factors underlying students’ attitudes towards translation technology, ought to be better understood. So far, all these remain unanswered.

To bridge the gap, we employ the motivational constructs of mindsets (Dweck, 2006; Lou and Noels, 2017) and future work self (Strauss et al., 2012; Taber and Blankemeyer, 2015) to investigate how these factors are related to students’ attitudes towards translation technology. The reasons for such a choice were two-fold. Firstly, with a long-standing presence in motivation research, both have proved crucial variables influencing students’ learning behaviors. To illustrate, mindsets which concern one’s beliefs about the malleability of their cognitive ability (Dweck, 2006), literally condition students’ goal orientation and actual efforts invested in learning (Husman and Lens, 1999; Lou and Noels, 2017, 2020). Future work self which represents the self-concept associated with one’s future aspiration (Strauss et al., 2012), directly affects students’ goal-driven and self-directed learning process (Guan et al., 2014; Zhang et al., 2016). Given the motivational significance mentioned above, the two guiding concepts may provide a valuable theoretical and practical framework for relevant measurement. Secondly, potential correlations have been shown between students’ mindsets, future work self, and attitudes towards translation technology. While these links are speculative and have not been empirically scrutinized, previous studies on computer-assisted language learning have already revealed that mindsets have far-reaching impacts on how students perceive technology-mediated learning (Zarrinabadi et al., 2022) and that future work self can play an antecedent role in predicting students’ interaction with technology (Coetzee, 2019). Consequently, the question could be raised as to whether these two constructs have the potential to predict students’ attitudes regarding translation technology adoption.

Therefore, the present paper reports on an initial effort to fill the gap through a questionnaire-based survey of 108 students studying on MTI Programs from three selected Chinese universities. This study aims to explore students’ attitudes towards translation technology in the Chinese context and examine how translation mindsets and future work self are related to it. The results serve as the preliminary step to understanding the current attitudes of Chinese student translators and the potential reasons why students embrace or reject translation technology in their learning or use. These, in turn, can help to inform TTT instructional designs, thus offering important implications for teaching translation technology in China and possibly elsewhere.

## 2. Theoretical framework

### 2.1. Attitudes towards translation technology

Translation technology functions as the umbrella term for a variety of technologies increasingly integrated into the translation process (Man et al., 2020). In the context of training of translation, technology seems to be the elephant in the room (Cheung, 2022). Given its wide application across the industry, training students in technology has become an indispensable part of translation education (Yan and Wang, 2022). Concerning TTT, according to Alcina Caudet (2002), students’ attitudes towards translation technology should be one of the essential didactic foci.

Attitudes are feelings about a particular thing or behavior (Ajzen, 1991; Lai et al., 2022). Accordingly, in the current study, students’ attitudes towards translation technology concern their perceptions towards using technology when they learn or do translation in and out of class. The prevailing viewpoint in research on attitudes constructs is to decompose them into affective and cognitive components (Wenden, 1991; Liaw, 2002). Although there still lacks clearly defined constructs for attitudes towards translation technology, we can combine the existing CALL attitude model (Vandewaetere and Desmet, 2009) and the TTTC model (He and Tao, 2022) as the framework for understanding. The CALL attitude model by Vandewaetere and Desmet (2009) mainly deals with the affective dimension. It is a multi-factor structure comprising attitudes toward the effectiveness of CALL, teacher influence and exhibition to CALL, etc. (Vandewaetere and Desmet, 2009; Zarrinabadi et al., 2022). The TTTC model by He and Tao (2022) mainly concerns the cognitive dimension. It echoes the call for “mindful technology” raised by Varlotta (2018), emphasizing that students should be self-aware to apply analytical, evaluative, and creative thinking when using technologies (He and Tao, 2022).

By integrating these two models, this study first defines the operational constructs of attitudes towards translation technology. It comprises four sub-scales, namely attitudes towards the effectiveness of translation technology, exhibition to translation technology, teacher influence, and mindfulness about translation technology. To be more specific, attitudes towards the effectiveness of translation technology are associated with the extent to which students perceive it as effective and useful for their translation or translation learning (Vandewaetere and Desmet, 2009). Exhibition to translation technology refers to the degree of exhibition or inhibition students feel when they learn and use translation technology (Vandewaetere and Desmet, 2009). Teacher influence concerns the extent to which teachers’ passion or encouragement influences students’ perceptions toward translation technology (Vandewaetere and Desmet, 2009). Mindfulness about translation technology denotes the degree to which students can be aware of applying translation technology critically and creatively (He and Tao, 2022).

As mentioned above, one key objective of technical competence training is stimulating students’ positive attitudes towards translation technology (Rico, 2017; Sánchez Ramos, 2022). Nevertheless, the status quo is that some students may bear negative preconceptions or even resistance since they view themselves as not computer savvy (Ehrensberger-Dow and O’Brien, 2015; Krüger, 2021). In such a sense, it is vital to help students overcome the attitudinal barriers in TTT. To this end, this study examines students’ attitudes towards translation technology and its underlying motivational antecedents.

### 2.2. Translation mindsets

The notion of mindsets has been the subject of research in various areas of educational psychology. As a crucial motivational construct, mindsets pertain to one’s beliefs about the malleability of cognitive abilities (Dweck, 2006). There are two clear patterns of mindsets for individuals: growth mindsets, which concern the belief that one’s cognitive abilities are malleable and can be improved through efforts, and fixed mindsets, which relate to the belief that one’s cognitive abilities are stable and cannot be changed easily (Dweck, 2006). Mindsets are domain-specific (Mercer and Ryan, 2010). For instance, language mindsets are the mindsets in second-language learning, denoting students’ beliefs in their language abilities (Lou and Noels, 2017, 2020; Papi et al., 2021; Zarrinabadi et al., 2022). Accordingly, in the current study, translation mindsets, which point to the mindsets in translation learning, can be defined as students’ beliefs in their abilities to learn and do the translation. Likewise, student translators usually have two primary translation mindsets. Students with growth translation mindsets believe their translation competence is changeable and can be improved by persistent practice. In contrast, those with fixed translation mindsets believe their translation competence cannot be altered, even through hard work.

Research indicated that students’ perceptions and adoptions of specific tasks could have roots in their mindsets (Dweck and Leggett, 1988). Following this, several studies have examined language mindsets regarding students’ attitudes towards technology-mediated language learning (TMLL). These studies showed that students with growth language mindsets are highly motivated and adaptive in TMLL (Waller and Papi, 2017; Rahimi and Zhang, 2022; Zarrinabadi et al., 2022), while those with fixed language mindsets feel less motivated and are maladaptive (Lou and Noels, 2017; Papi et al., 2021). Finally, the research findings have revealed the antecedent role of growth language mindsets in students’ positive attitudes towards TMLL and cautioned against the negative impacts of fixed language mindsets on this front (Zarrinabadi et al., 2022). Based on these discoveries, we expected translation mindsets to be an essential predictor of students’ attitudes towards translation technology. Furthermore, we predicted that growth translation mindsets would positively correlate with students’ attitudes towards translation technology, and fixed translation mindsets would associate with it negatively. Hence, one purpose of this study is to investigate and validate this connection within the Chinese MTI context.

### 2.3. Future work self

Future work self represents an aspect of the self-concept associated with the individual’s future career aspirations and expectations (Strauss et al., 2012). It is introduced to better examine the motivation behind students’ proactive learning behaviors (Strauss et al., 2012; Guan et al., 2014). As an internal link between self-concept and future hopes, this concept can function as the incentive to enable students to strive toward their imagined future (Lu, 2020). According to the existing literature (Strauss et al., 2012; Zhang et al., 2016; Ling et al., 2022), future work self mainly comprises two dimensions: future work self-salience and future work self-elaboration. Specifically, future work self-salience, which denotes the quality aspect of future work self, refers to the degree to which the individual’s future representation is clear (Strauss et al., 2012). The more salient the future work self is, the better it can activate students’ self-concept (Strauss et al., 2012; Ling et al., 2022). Future work self-elaboration, which concerns the description aspect of future work self, pertains to the degree of detailed planning and description of their future representation (Zhang et al., 2016). Students with elaborate future work self are more likely to make elaborate plans aligned with their future goals (Lu, 2020).

In the context of technological education, prior research has shown that a salient and elaborate future work self-prompts students’ self-directed learning of technology aimed at self-development (Hoyle and Sherrill, 2006; Ngampornchai and Adams, 2016). When capturing current and future self-discrepancies, the student with a high-level future work self would be highly motivated to cultivate positive psychology for sound interactions with technology (Ardies et al., 2015; Lu, 2020). On this basis, numerous studies empirically confirmed the positive correlations between students’ future work self and their psychological strengths in technological learning (Tucker, 2014; Ardies et al., 2015; Taghizadeh and Hajhosseini, 2021). In such a sense, this notion gains substantial relevance in TTT. In light of the above findings, we proposed that future work self might affect students’ attitudes towards translation technology. The two variables were expected to be related positively. Thus, set in the Chinese MTI context, the present study also seeks to better understand how this motivational antecedent underlines students’ attitudes towards translation technology.

## 3. Research questions and hypotheses

Accordingly, two primary research questions were formulated in the current study:

1. What are students’ attitudes towards translation technology (in the Chinese MTI context)?
2. How do translation mindsets (growth translation mindsets and fixed translation mindsets) and future work self (future work self-salience and future work self-elaboration) relate to students’ attitudes towards translation technology?

**Figure 1:**
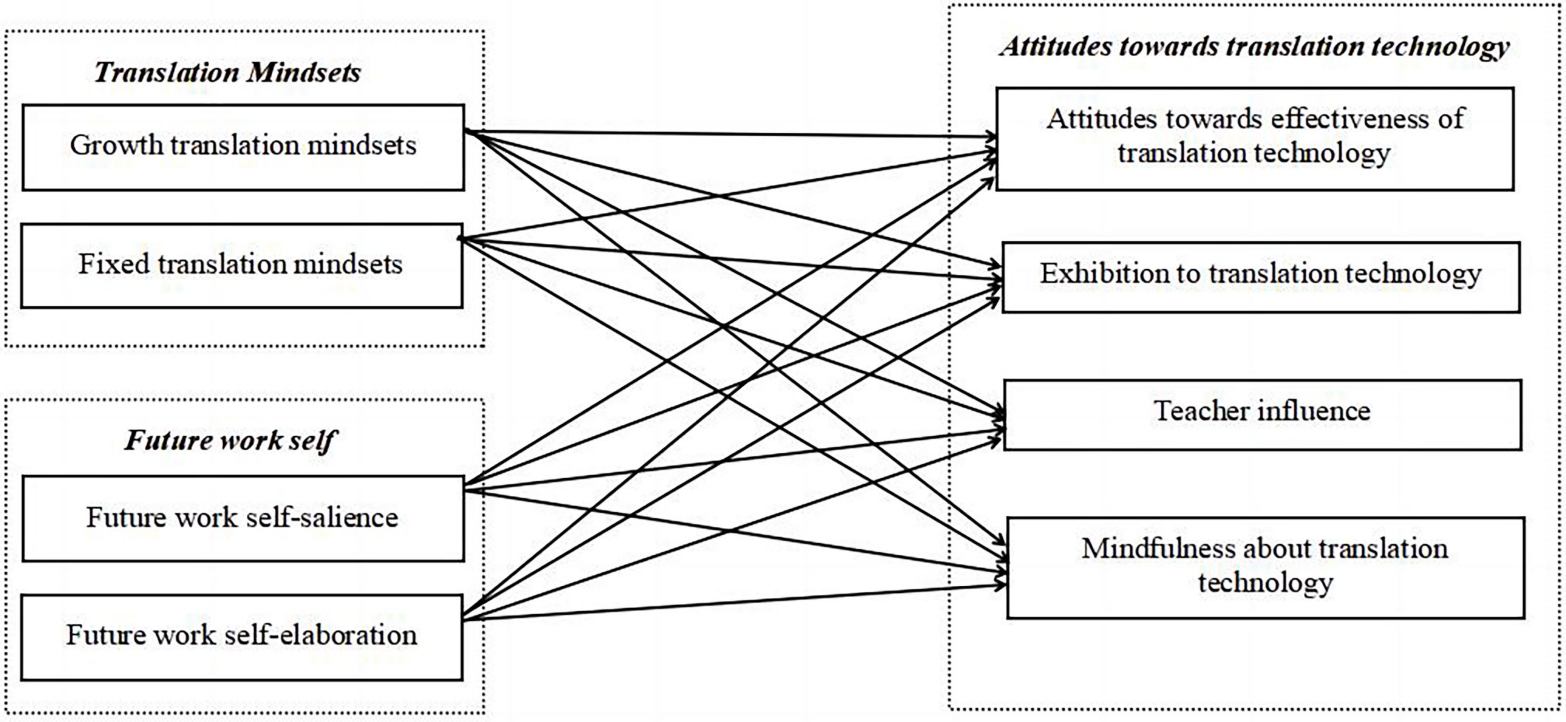
Hypothesized research model for RQ2.

Given the scope, this is a descriptive and exploratory study. The descriptive study tries to reveal students’ attitudes towards translation technology in the Chinese MTI context (RQ1). The exploratory study investigates the underlying motivational antecedents in relation to it (RQ2). As discussed above, students’ differences in translation mindsets and future work self might correspond to different attitudes towards translation technology. In other words, some structural correlations may exist between the above variables. According to the theoretical literature reviewed earlier, the study proposed the following hypotheses for RQ2. Figure 1 is the hypothesized research model, which mirrors all the hypotheses.

In this research, we proposed that growth translation mindsets would positively predict attitudes towards translation technology. As indicated by the previous research, students with growth mindsets would develop positive beliefs and invest more effort in both traditional and technology-mediated learning (Lou and Noels, 2017; Khajavy et al., 2021). Therefore, in the context of TTT, they are expected to perceive translation technology as effective and instrumental, be encouraged by the teacher, feel less inhibited, and develop an awareness of critical application. In contrast, we also anticipated that fixed translation mindsets would be negatively connected with attitudes towards translation technology. As confirmed by past studies, students with fixed mindsets tend to feel more anxious and see less use in TMLL (Lou and Noels, 2017; Zarrinabadi et al., 2022). Predictably, in TTT, they would hardly be influenced by the teacher, find little value in translation technology, fail to be creative, and feel more inhibited when using it.

Furthermore, we hypothesized that future work self would positively predict attitudes towards translation technology. As proved by the extant literature, in the learning context (including TMLL), a salient future work self could be a positive psychological resource driving students towards their desired future (Strauss et al., 2012; Lu, 2020). An elaborate future work self could prompt detailed planning or schema, facilitating sound interactions between students and the surroundings (Strauss et al., 2012; Ling et al., 2022). Thus, in the situation of TTT, the student with a high-level future work self would consider translation technology as effective, see value in it, show exhibition to it, bear the awareness of critical and creative use, and interact well with the teacher.

## 4. Methodology

### 4.1. Context

The current research was conducted in the Chinese MTI (Master of Translation and Interpreting) context. Since its launch by the Chinese Academic Degree Committee in 2007, 316 universities nationwide have set up MTI programs. Under the guidance of the Chinese National Advisory Committee for MTI Training, these programs aim to train high-level and professional translators and interpreters for the ever-demanding market (Tao, 2019). In response to the technological turn in the language service industry, more and more Chinese institutes have begun to incorporate translation technology into their MTI curriculum, a move required by 2011 Revised Edition of Recommended Program Plan. According to a survey conducted by Wang et al. (2018), 224 out of 249 (90.4%) MTI programs in China offer courses related to translation technology (mainly during the first year of training). However, despite the widespread passion for TTT in China, there are some acute problems: for example, insufficient attention paid to TTT, lack of teaching resources, lack of eligible TTT teachers, and notably, students’ mixed perceptions towards translation technology (Wang et al., 2018). Among them, the question of “how to develop students’ positive attitudes in TTT” needs to be investigated. Thus, this study seeks to examine students’ attitudes towards translation technology and the two motivational antecedents related to it, namely the translation mindsets and future work self.

### 4.2. Participants

Participants of the present research were chosen by purposive stratified sampling (You and Dornyei, 2016; Man et al., 2020). For this purpose, three geographical strata were considered: northwest, central, and south regions. Finally, 108 grade 2021 MTI students from three selected Chinese universities in the above regions participated in this study. Table 1 presents the descriptive profiles of the selected universities. As shown below, the three chosen institutes are Ningxia University (situated in the northwest of China), Central South University (situated in central China), and Hainan University (situated in the south of China). All are on the list of Double First-Class universities (the higher education development plan initiated by the Chinese government in 2015 to develop elite Chinese universities into world-class ones by 2050). All offer one or two (even three) translation technology courses to students in their MTI programs.

**Table 1:** Descriptive profiles of the selected universities.

Selected universities	Description
Ningxia university	situated in the northwest of China, listed in Double First-Class universities, operating a 2-year MTI program, offering two translation technology courses (CAT/Advanced CAT, 32 teaching hours each) during the first year of training.
Central South university	situated in central China, listed in Double First-Class universities, operating a 3-year MTI program, offering three translation technology courses (CAT/Corpus-based Translation Studies /Introduction to translation technology, 32 teaching hours each) during the first year of training.
Hainan university	situated in the south of China, listed in Double First-Class universities, operating a 3-year MTI program, offering one translation technology course (CAT, 32 teaching hours) during the first year of training.

Table 2 shows the demographic characteristics of participants from the selected universities. All available grade 2021 MTI students were recruited. Interpreting students were also included because they also learn and use translation technology for their translation and interpreting work. They had similar language backgrounds: they were Chinese and learned English as their second language. All have passed TEM-8, the test to measure English proficiency for English-majored undergraduates in China. When the study was initiated, all participants had received formal training in translation technology through the relevant courses offered by their home institutes. As displayed below, there were 86 (79.6%) female students and 22 (20.4%) male students. They were in their early twenties, with seven (6.5%) aged 22, 54 (50%) aged 23, and 47 (43.5%) aged 24. Forty-five (41.6%) were chosen from Ningxia University, 30 (27.8%) from Central South University, and 33 (30.6%) from Hainan University. Among them, 84 (77.8%) specialized in translation, and 24 (22.2%) specialized in interpreting. All students were informed of the study’s purpose and participated voluntarily and anonymously. Ethical approval was also obtained from the ethics committees of the selected universities.

**Table 2:** Demographic characteristics of the participants.

	N	%
Gender		
Male	22	20.4
Female	86	79.6
Total	108	100
Age		
22	7	6.5
23	54	50
24	47	43.5
Total	108	100
Participating university		
Ningxia university	45	41.6
Central South university	30	27.8
Hainan university	33	30.6
Total	108	100
Major		
Translation	84	77.8
Interpreting	24	22.2
Total	108	100

### 4.3. Instrument

To address the research questions, we jointly used attitudes towards translation technology scale (ATTS), translation mindsets inventory (TMI), and future work self-scale (FWSS; Table 3). In this study, these survey instruments were administered in the format of one combined questionnaire (10.6084/m9.figshare.21966881). This questionnaire consisted of two parts. The first part solicited some demographic information from students (e.g., university, age, gender, and major). The second part included 26 items measuring students’ attitudes towards translation technology, translation mindsets, and future work self. All items were measured on a 6-point Likert scale, with 1 indicating “strongly disagree” and 6 showing “strongly agree.” The 6-point Likert scale was used to encourage students to consider the items more carefully and make either positive or negative choices (Taherdoost, 2019).

**Table 3:** Measure instruments.

Research questions	Instruments	Purpose
RQ1	Attitudes towards translation technology scale (ATTS)	To describe students’ attitudes towards translation technology in the Chinese MTI context.
RQ2	Attitudes towards translation technology questionnaire (ATTS) Translation mindsets inventory (TMI) Future work self-scale (FWSS)	To explore the structural relations between students’ translation mindsets, future work self, and their attitudes towards translation technology.

To answer RQ1, or rather to describe students’ attitudes towards translation technology in the Chinese MTI context, ATTS with 12 items was utilized in the second part of the combined questionnaire. This scale was developed based on the CALL attitude questionnaire (Vandewaetere and Desmet, 2009; Zarrinabadi et al., 2022) and the TTTC model (He and Tao, 2022). Some items were adapted to relate to the context of TTT and the work of student translators. Specifically, nine items adapted from the CALL attitude questionnaire (Vandewaetere and Desmet, 2009; Zarrinabadi et al., 2022) were employed to measure attitudes towards the effectiveness of translation technology (3 items), exhibition to translation technology (3 items), and teacher influence (3 items). Three items adapted from the TTTC model (He and Tao, 2022) were adopted to measure students’ mindfulness about translation technology. Sample items included “learning translation technology is valuable and useful” (attitudes towards effectiveness of translation technology), “I do not experience anxiety when trying to use translation technology” (exhibition to translation technology), “Teachers’ attitudes towards translation technology largely define my attitudes towards it” (teacher influence) and “When learning translation technology, I want to know more than just how to apply it” (mindfulness about translation technology). All items had a 6-point Likert scale survey. The Cronbach’s alpha of the four sub-scales was between 0.751 and 0.854, exceeding the minimum threshold of 0.70 recommended by Hair et al. (2006).

To answer RQ2, or rather to explore the structural relations between students’ translation mindsets, future work self, and their attitudes towards translation technology, ATTS, TMI and FWSS were collectively used in the second part of the combined questionnaire. As elaborated above, ATTS was employed to measure students’ attitudes towards translation technology. In addition, an eight-item TMI (Papi et al., 2019, 2022) and a six-item FWSS (Strauss et al., 2012; Lu, 2020) were applied to assess students’ translation mindsets and future work self, respectively. To illustrate, we developed TMI based on Papi et al. (2019, 2021) eight-item language mindsets inventory. The item statements were simply adapted to accord with the domain of translation learning, targeting students’ perceptions about the malleability of their translation competence. Among them were four growth translation mindsets items and four fixed translation mindsets items, with the response scale ranging from 1 (strongly disagree) to 6 (strongly agree). Sample items included “in translation learning, if you work hard, you will always get better” (growth mindset) and “you have a certain amount of translation competence, and you cannot do much to change it” (fixed mindset). Cronbach’s alpha value recorded 0.793 for growth translation mindsets and 0.739 for fixed translation mindsets. FWSS designed and validated by Strauss et al. (2012) was utilized to assess future work self. The six-item FWSS evaluated two aspects on a 6-point Likert scale: three items were used to measure future work self-salience, and another three were used to survey future work self-elaboration. Sample items were “I am very clear about who and what I want to become in my future” (future work self-salience) and “I am planning what I want to do in the next few years of my future” (future work self-elaboration). FWSS has already demonstrated good reliability and validity in numerous studies (Strauss et al., 2012; Lu, 2020; Ling et al., 2022). In this study, Cronbach’s alpha for these two constructs was 0.868 and 0.862.

As noted above, all three scales used in the second part of the combined questionnaire (with 26 items) represent rigorous design, good reliability, and firm theoretical grounding. Furthermore, two experts in TTT reviewed the content validity of all adapted items. A detailed list of the scales and measurement constructs in this study is presented in Table 4.

**Table 4:** List of the scales and measurement constructs.

Construct	Abbr.	Descriptions	Items	Scales & references
Attitudes towards effectiveness of translation technology	AETT	The extent to which students perceive translation technology as effective and useful for their translation or translation learning.	3	**ATTS**; Vandewaetere and Desmet (2009); Zarrinabadi et al. (2022)
Exhibition to translation technology	ETT	The degree of the exhibition or inhibition that students feel when they learn and use translation technology.	3	
Teacher influence	TI	The extent to which teachers’ passion or encouragement influences students’ perceptions towards translation technology.	3	
Mindfulness about translation technology	MTT	The degree to which students can be aware of applying translation technology critically and creatively.	3	He and Tao (2022)
Growth translation mindsets	GTM	The belief that their translation competence is changeable and can be improved by persistent practice.	4	**TMI**; Papi et al. (2019, 2021)
Fixed translation mindsets	FTM	The belief that their translation competence cannot be altered, even through hard work.	4	
Future work self-salience	FWSS	The degree to which the individual’s future representation is clear.	3	**FWSS**; Strauss et al. (2012)
Future work self-elaboration	FWSE	The degree of detailed planning and description of their future representation.	3	

### 4.4. Data collection procedure

The data were collected in September of 2022 using a paper questionnaire survey. The researchers administered this combined questionnaire during their separate visits to the selected universities. They tried to approach all available grade 2021 MTI students in these institutes. At this time, these students have already taken translation technology course(s) during their first-year MTI studies.

Specifically, on September 11th, 2022, one of the researchers visited Ningxia University. After receiving approval from the ethics committee of the School of Foreign Languages and Cultures, she gathered all available grade 2021 MTI students who were willing to participate in one classroom with the help of the teaching fellows here and delivered the paper questionnaire to them. Before completing the questionnaire, the survey purposes were clarified, and the targeted students were assured of their rights to anonymity and confidentiality. The definition and the main types of translation technology have also been discussed with the participating students. No new variables were introduced. Moreover, to improve their response accuracy, the concept of translation mindsets and future work self was introduced, and all 26 items were also explained. It took the students approximately 12 min to finish the questionnaire. Altogether, the whole investigation process took around 30 min. Similar survey proceedings also took place at Hainan University on September 22nd, 2022, and Central South University on September 29th, 2022. By the end of September 2022, 123 responses were collected from grade 2021 MTI students in the selected universities. Three incomplete ratings were excluded. Twelve univariate and multivariate outliers were identified (using standard score and Mahalanobis Distance test) and dropped (Tabachnick and Fidell, 2018). The exclusion of 15 invalid questionnaires provided a final size of 108.

### 4.5. Data analysis

The data collected were statistically analyzed in accordance with the research questions. To answer RQ1, data collected from ATTS in the combined questionnaire were analyzed using SPSS 22. A descriptive analysis was performed to calculate the means for the 4 s-order constructs (AETT, TI, ETT, and MTT) and the first-order indicator (ATT). Skewness and Kurtosis data were also included to examine the normal distribution. Furthermore, the one-sample t-test was conducted to compare the above means with the test value of 4 and 5, with a view to depicting students’ overall ATT level.

To answer RQ2, a two-step SEM (structural equation modeling) approach recommended by Anderson and Gerbing (1988) was adopted to analyze the data collected from ATTS, TMI, and FWSS. Specifically, confirmatory factor analysis was first performed to check the scales’ reliability, convergent validity, and discriminant validity (Kline, 2015). Reliability was measured by the index of Cronbach’s alpha (higher than 0.7) and composite reliability (CR, higher than 0.7; Hair et al., 2006). Convergent validity was evaluated through factor loadings (higher than 0.6) and average variance extracted (AVE, higher than 0.5) estimates (Hair et al., 2006; Byrne, 2013). Discriminant validity was assessed by testing whether the AVE square roots of each construct were greater than its bivariate correlation coefficients with other constructs (Fornell and Larcker, 1981; Esteban-Millat et al., 2018). Then the structural model was executed to test the proposed hypotheses and examine the correlations between variables. As recommended by the previous research (Bagozzi and Yi, 1988; Kline, 2005, 2011; Lin, 2011; Byrne, 2013), a combination of model fit indices such as Normed Chi-square (x^2^/df, lower than 3), Comparative Fit Index (CFI, higher than 0.9), Goodness-of-fit Index (GFI, higher than 0.8), Adjusted Goodness-of-fit Index (AGFI, higher than 0.8) and Root Mean Square Error of Approximation (RMSEM, lower than 0.08) were used. The whole SEM analysis was conducted in SPSS 22 and AMOS 26.

## 5. Results

### 5.1. Students’ attitudes towards translation technology

**Figure 2:**
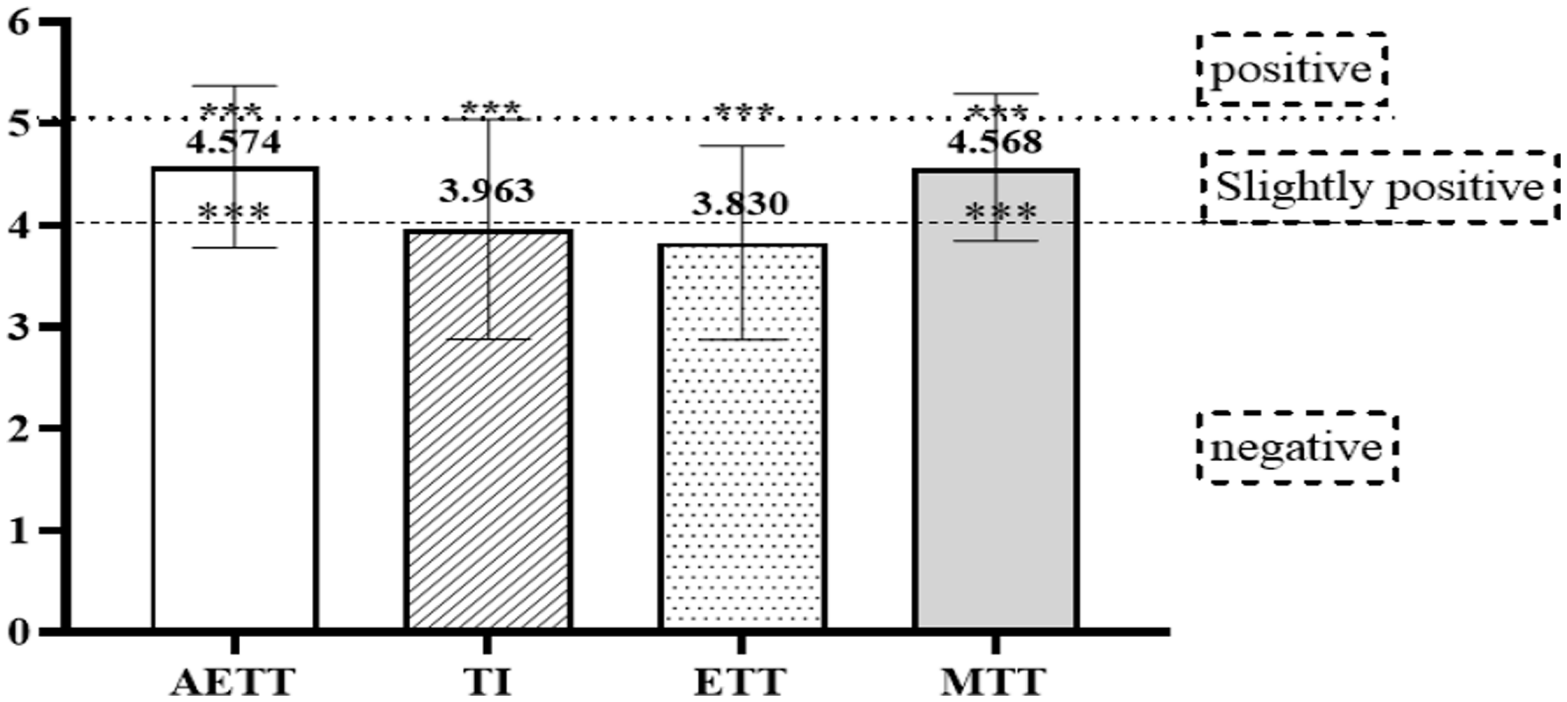
Means and one-sample *t*-test results of four ATT second-order constructs. *p<0.05; **p<0.01; ***p<0.001.

RQ1 aims to describe students’ attitudes towards translation technology (ATT) in the Chinese MTI context. As elaborated above, to address this question, participants were asked to rate their agreement or disagreement with 12 statements on the 6-point ATTS in the combined questionnaire. The higher ratings imply more positive attitudes, with 4 indicating slightly positive ATT, 5 positive ATT, and 6 strongly positive ATT (Doherty et al., 2011). The statistical details are displayed in Figure 2 and Table 5.

**Table 5:** Descriptive statistics and one-sample *t*-test results of ATT constructs.

ATT constructs	Means	SD	Skewness	Kurtosis	One sample *t*-test (test value = 4)	One sample *t*-test (test value = 5)
AETT	4.574	0.796	−0.420	0.248	0.000	0.000
TI	3.963	1.080	−0.384	−0.277	0.722	0.000
ETT	3.830	0.954	0.013	−0.360	0.067	0.000
MTT	4.568	0.721	−0.433	0.461	0.000	0.000
**ATT**	**4.180**	**0.625**	**0.363**	**0.403**	**0.003**	**0.000**

As shown in Table 5, all skewness and kurtosis values are within the desirable range (Kim, 2013), verifying the data’s normality assumption. Means for the 4 s-order construct range from 3.830 to 4.574, implicating that students’ overall attitudes towards translation technology are slightly positive. The one-sample t-test results again confirmed this (see Figure 2; Table 5).

More specifically, AETT mean scores the highest rating (M = 4.574, SD = 0.796), which is statistically different both from the test value 4 (slightly positive attitudes) and 5 (positive attitudes; see Figure 2; Table 5). These results suggest that Chinese MTI students perceive translation technology as slightly effective. In other words, given the increasing popularity of translation technology in the industry and involving of TTT in Chinese translation education, MTI students in China have already developed the belief that translation technology could exert some positive effects on their translation. However, they have failed to capture its total value and evolve positive attitudes towards its effectiveness so far.

MTT mean is 4.568 (SD = 0.721), also ranging between 4 and 5 and statistically differing from them (see Figure 2; Table 5). The results reveal that Chinese MTI students are slightly mindful of translation technology at the current time. That is to say, when learning and using translation technology, MTI students in China now do have a slight awareness of involving reasoning and reflection in its application. Nevertheless, they have failed to develop adequate “analytical, synthetic and holistic thinking” towards translation technology (He and Tao, 2022) and, in turn, become mindful of it.

TI mean stands at 3.963 (SD = 1.080), with no significant differences found between it and the test value 4 (see Figure 2; Table 5). These results indicate that Chinese MTI students’ attitudes towards translation technology are only slightly influenced by their teachers in the TTT context. This is somewhat surprising because teacher effects on students’ learning attitudes and behaviors have already been proven to be substantial by numerous studies (Ardies et al., 2015; Blazar and Kraft, 2017; Blazar, 2018). One possible explanation is the lack of qualified translation technology teachers in China. According to the industrial survey by the Translators Association of China in 2018, only 8.04% of TTT teachers were quite familiar with the translation technology (Wang et al., 2018).

The ETT mean is also below 4 (M = 3.830, SD = 0.954), with no statistical difference (see Figure 2; Table 5). The results demonstrate the slight exhibition Chinese MTI students feel towards translation technology. To put it in another way, MTI students in China still feel inhibited or experience some anxiety when trying to learn and use translation technology. This finding is in line with the previous studies suggesting the cognitive resistance encountered by students when engaging with TTT (O’Brien, 2012; Ehrensberger-Dow and O’Brien, 2015).

The mean value of the first-order indicator ATT reports 4.180 (SD = 0.625), statistically different from the test values 4 and 5 (see Table 5). Combined with the descriptive analysis of the above 4 second-order constructs, we could tentatively conclude that Chinese MTI students’ overall attitudes towards translation technology are slightly positive. Right now, they perceive translation technology to be slightly effective for translation and are slightly mindful of it; they are only slightly influenced by their teachers and still feel inhibited when trying to learn and use it.

### 5.2. Structural relations between students’ TM, FWS and ATT

RQ2 seeks to explore the structural relations between students’ translation mindsets (TM), future work self (FWS), and their attitudes towards translation technology (ATT). As illustrated above, to answer this question, participants were asked to rate their agreement or disagreement with 26 statements on the 6-point ATTS, TMI, and FWSS in the combined questionnaire. With the data collected, we first conducted data screening and preliminary descriptive statistics using SPSS 22. Then, a two-step SEM approach (confirmatory factor analysis of the measurement model and structural equation modeling of the structural model) was employed using AMOS 26 for the structural analysis and hypotheses testing (Anderson and Gerbing, 1988).

#### 5.2.1. Preliminary descriptive analysis

A total of 123 responses were collected from grade 2021 MTI students in the selected universities. The collected data were first screened and cleaned. One hundred and eight valid responses were maintained for data analysis.

Table 6 shows each construct’s minimum, maximum, means, standard deviations, variance, skewness, and kurtosis. Their Skewness and Kurtosis values all fall within the recommended range, suggesting that the normality assumption was satisfied (Kim, 2013). Their means range between 3.718 (SD = 0.948) and 4.574 (SD = 0.796), all scoring below 5. To be specific, AETT has the highest mean value (4.574 ± 0.796), followed by MTT (4.568 ± 0.721), FWSE (4.321 ± 0.923), GTM (4.245 ± 0.900), TI (3.963 ± 1.080), ETT (3.830 ± 0.954), FWSS (3.775 ± 1.017), and FTM (3.718 ± 0.948), respectively. These descriptive statistics indicate that students’ overall responses to ATT, TM, and FWS are not that positive. The relatively low ratings of all the constructs likely illustrate the motivational antecedent role of translation mindsets and future work self in students’ attitudes towards translation technology.

**Table 6:** Descriptive analysis of each construct.

Constructs	Minimum	Maximum	Means	SD	Variance	Skewness	Kurtosis
AETT	2.330	6.000	4.574	0.796	0.633	−0.420	0.248
TI	1.000	6.000	3.963	1.080	1.166	−0.384	−0.277
ETT	1.670	6.000	3.830	0.954	0.911	0.013	−0.360
MTT	2.670	6.000	4.568	0.721	0.520	−0.433	0.461
GTM	1.000	6.000	4.245	0.900	0.811	−1.017	1.463
FTM	1.250	6.000	3.718	0.948	0.900	−0.218	−0.319
FWSS	1.670	6.000	3.775	1.017	1.034	−0.026	−0.943
FWSE	1.000	6.000	4.321	0.923	0.851	−1.033	1.531

#### 5.2.2. Measurement model assessment

Prior to the structural modeling, CFA (confirmatory factor analysis) was adopted to assess the quality of the measurement model. All eight constructs were examined regarding their reliability, convergent validity, and discriminant validity (Kline, 2015). The reliability and validity results of the measurement model are shown in Tables 7, 8.

**Table 7:** Reliability and convergent validity results.

Constructs	Items	Cronbach’s alpha	Factor loadings	CR	AVE
AETT	AETT1	0.862	0.702	0.749	0.499
	AETT2		0.73		
	AETT3		0.687		
TI	TI1	0.854	0.796	0.858	0.668
	TI2		0.831		
	TI3		0.824		
ETT	ETT1	0.753	0.618	0.759	0.515
	ETT2		0.777		
	ETT3		0.747		
MTT	MTT1	0.781	0.664	0.756	0.508
	MTT2		0.698		
	MTT3		0.685		
GTM	GTM1	0.793	0.69	0.799	0.499
	GTM2		0.753		
	GTM3		0.741		
	GTM4		0.635		
FTM	FTM1	0.739	0.668	0.803	0.511
	FTM2		0.675		
	FTM3		0.742		
	FTM4		0.604		
FWSS	FWSS1	0.868	0.722	0.873	0.698
	FWSS2		0.878		
	FWSS3		0.896		
FWSE	FWSE1	0.862	0.812	0.862	0.676
	FWSE2		0.796		
	FWSE3		0.858		

**Table 8:** Discriminant validity results.

Constructs	MTT	ETT	TI	AETT	FWSE	FWSS	FTM	GTM
MTT	**0.713**							
ETT	0.294	**0.718**						
TI	0.311	0.373	**0.817**					
AETT	0.63	0.213	0.471	**0.706**				
FWSE	0.364	0.06	0.252	0.335	**0.822**			
FWSS	0.468	0.225	0.369	0.436	0.541	**0.835**		
FTM	−0.052	−0.162	−0.312	−0.045	−0.004	0.224	**0.715**	
GTM	0.466	0.37	0.396	0.362	0.328	0.406	0.115	**0.706**

Square roots of AVE are presented in bold on diagonals.

Table 7 reports the results of the reliability and convergent validity of the measurement model. As shown in it, values of composite reliability (ranging between 0.749 and 0.873) and Cronbach’s Alpha (ranging between 0.739 and 0.868) of the eight constructs are all above the recommended threshold of 0.7, indicating good reliability. Additionally, factor loadings of all items are between 0.604 and 0.896, all higher than the acceptable criterion of 0.6. All average variance extracted values exceed 0.5, except for the construct of AETT and GTM (both 0.499, very close to the minimum level). According to Fornell and Larcker (1981), it is still acceptable if the construct AVE is between 0.4 and 0.5, but its composite reliability is higher than 0.6. Hence, the convergent validity is adequate in the current study. Table 8 displays the correlation matrix and discriminant validity results. As demonstrated, ranging from 0.713 to 0.812, all the constructs’ AVE square root values are higher than their corresponding correlation coefficients with other constructs. These results prove that the discriminant validity is statistically present.

Taken together, the values of all indicators in this study meet their recommended criteria. Hence, the overall reliability and validity of the measurement model are confirmed and validated.

#### 5.2.3. Structural equation modeling

**Figure 3:**
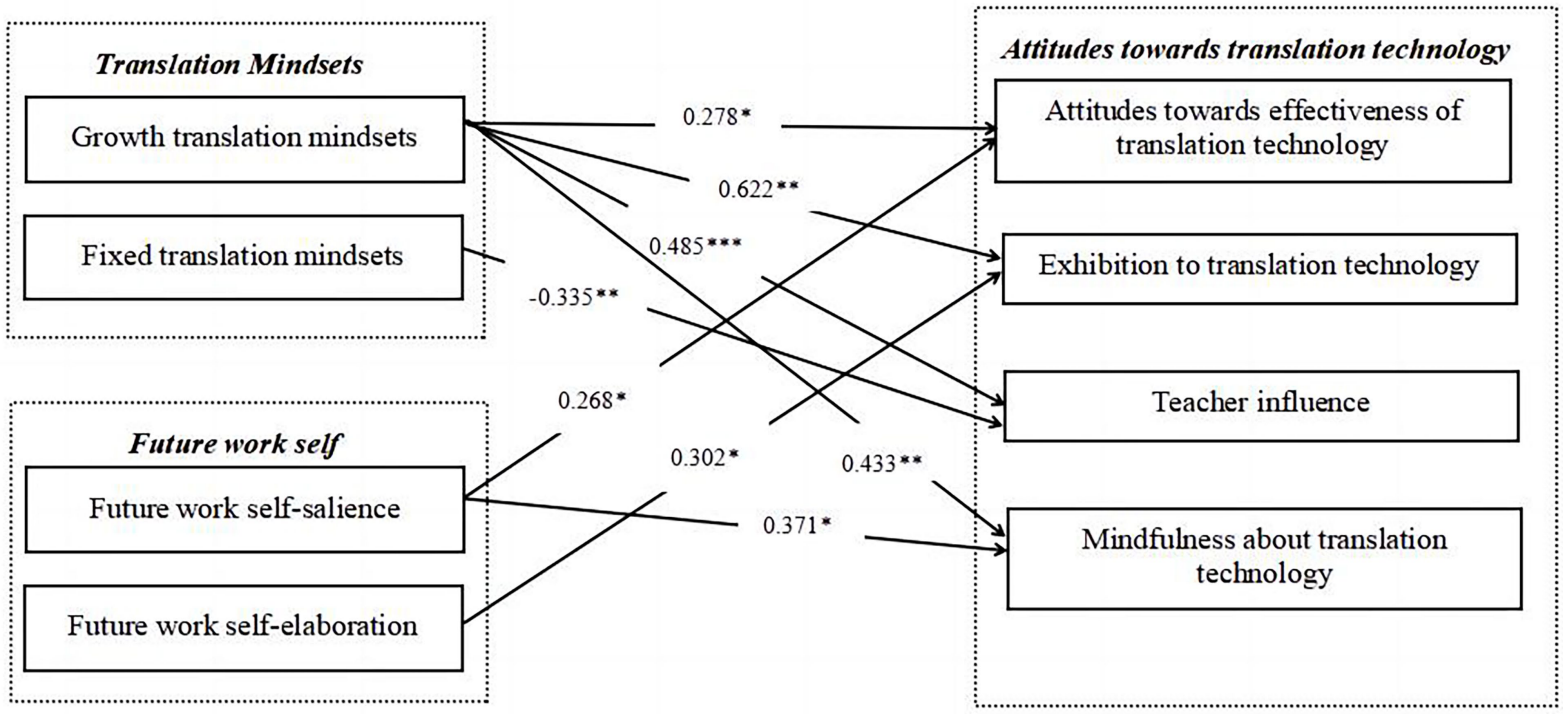
The modified structural model for relations between students’ TM, FWS and ATT.

To explore the structural relations between students’ translation mindsets (TM), future work self (FWS), and their attitudes towards translation technology (ATT), the hypothesized research model (Figure 1) was tested by AMOS 26. As seen in Figure 1, students’ motivational factors TM and FWS were considered to be the predictors of all the four sub-constructs of their ATT (AETT, TI, ETT, and MTT). All potential paths were incorporated into the hypothesized model. As illustrated above, five indices were chosen to assess the model fit according to the previous studies (Bagozzi and Yi, 1988; Kline, 2005, 2011; Lin, 2011; Byrne, 2013). The initial SEM results revealed a good fit: x^2^/df was 1.373 (< 3); GFI was 0.802 (> 0.8); AGFI was 0.789 (close to the recommended 0.8); CFI was 0.906 (> 0.9); and RMSEA was 0.059 (< 0.08). The initial results also suggested that eight out of 16 path coefficients were statistically significant with a value of p less than 0.05. On this basis, we removed all the non-significant paths and tested the modified model again. SEM results of the modified model are presented in Tables 9–11 and Figure 3.

**Table 9:** Model fit indices.

Fit indices	Value	Standard	Results
X^2^/df	1.375	< 3 (Hayduk, 1987; Kline, 2005)	Good
GFI	0.806	>0.8 acceptable; >0.9 good (Bagozzi and Yi, 1988; Kline, 2011)	Acceptable
AGFI	0.798	>0.8 acceptable; >0.9 good (Kline, 2011)	Very close to acceptable
CFI	0.907	>0.9 (Bagozzi and Yi, 1988; Kline, 2005)	Good
RMSEA	0.059	<0.08 (Byrne, 2013)	Good

**Table 10:** Parameter estimates of path analysis.

Causal path	Unstd.	S.E.	C.R.	*P*	Std.	*R* ^2^
AETT←GTM	0.269*	0.116	2.321	0.02	0.278	0.213
AETT←FWSS	0.193*	0.077	2.506	0.012	0.268	
TI←GTM	0.628***	0.169	3.709	***	0.485	0.378
TI←FTM	−0.443**	0.159	−2.786	0.005	−0.335	
ETT←GTM	0.52**	0.164	3.165	0.002	0.622	0.34
ETT←FWSE	0.233*	0.115	2.018	0.044	0.302	
MTT←GTM	0.312*	0.12	2.607	0.009	0.433	0.462
MTT←FWSS	0.199*	0.08	2.497	0.013	0.371	

Unstd. = non-standardized estimate; S.E. = standard error; C.R. = critical ratio; Std. = standardized estimate; *p < 0.05, **p < 0.01, ***p < 0.001.

**Table 11:** Regression weights.

	Unstd.	S.E.	C.R.	*P*	Std.
**Mindsets**					
GTM AETT	0.269*	0.116	2.321	0.02	0.278
GTM TI	0.628***	0.169	3.709	***	0.485
GTM ETT	0.52**	0.164	3.165	0.002	0.622
GTM MTT	0.312**	0.12	2.607	0.009	0.433
FTM TI	−0.443**	0.159	−2.786	0.005	−0.335
**Future work self**					
FWSS AETT	0.193*	0.077	2.506	0.012	0.268
FWSS MTT	0.199*	0.08	2.497	0.013	0.371
FWSE ETT	0.233*	0.115	2.018	0.044	0.302

*p < 0.05, **p < 0.01, ***p < 0.001.

Table 9 shows the fit indices of the modified model. All values of the indices fall within the acceptable range: x^2^/df = 1.375 (<3), GFI = 0.806 (>0.8), AGFI = 0.798 (very close to the acceptable level of 0.8), CFI = 0.907 (>0.9), and RMSEA = 0.059 (<0.08). Hence, the modified model also shows an excellent fit for the data.

Parameter estimates of the path analysis are reported in Table 10. As manifested in it, growth translation mindsets and future work self-salience positively influence AETT, indicating their role in encouraging students’ positive belief in the effectiveness of translation technology for translation. Growth translation mindsets and future work self-elaboration positively impact ETT, implying their utility in alleviating students’ inhibition and anxiety when learning and using translation technology. Growth translation mindsets and future work self-salience positively predict MTT, suggesting their value in arousing students’ awareness to apply translation technology critically and creatively. Moreover, the results also indicate that growth translation mindsets positively correlate with students’ teacher influence, whereas fixed translation mindsets negatively associate with it. Comparatively, growth translation mindsets, the individual intrinsic motivator in translation learning, exert the most decisive positive impact upon both AETT, TI, ETT, and MTT.

Table 11 presents the results in another way, displaying the corresponding regression weights of translation mindsets and future work self separately. Results regarding translation mindsets showcase that growth translation mindsets positively and significantly predict students’ attitudes towards the effectiveness of translation technology (Std. = 0.278, *p* = 0.02), teacher influence (Std. = 0.485, *p* < 0.001), exhibition to translation technology (Std. = 0.622, *p* = 0.002), and mindfulness about translation technology (Std. = 0.433, *p* = 0.009). Additionally, fixed translation mindsets are negatively and significantly related to students’ teacher influence (Std. = −0.335, *p* = 0.005). As for future work self, future work self-salience positively and significantly predicts students’ attitudes towards the effectiveness of translation technology (Std. = 0.268, *p* = 0.012) and mindfulness about translation technology (Std. = 0.371, *p* = 0.013). Furthermore, future work self-elaboration is positively and significantly related to students’ exhibition to translation technology (Std. = 0.302, *p* = 0.044). Thus, generally speaking, our initial hypotheses of their structural relations are partially supported in this study. Finally, the modified structural model of correlations between students’ translation mindsets, future work self, and attitudes towards translation technology is validated and presented in Figure 3.

## 6. Discussion

Through a questionnaire-based survey, the current study seeks to describe students’ ATT in the Chinese MTI context and explore how the motivational factors TM and FWS are related to it. Based on the results analyzed above, the following discussions are presented.

Regarding RQ1, the empirical data reveal slightly positive attitudes towards translation technology for Chinese MTI students. This result might be attributable to several reasons. First, students’ trust towards translation technology is still in question. Given its increasing incorporation into the industry and education, MTI students in China have already realized the importance of learning to use it. However, they may still be concerned about some TT flaws: for instance, producing various errors and unnatural expressions, impeding the selection efficiency, or undercutting their creativity, etc. (Briggs, 2018; Liu et al., 2022; Rico and Gonzalez Pastor, 2022). These mixed perceptions may contribute to their belief that translation technology is only slightly effective for their translation, and they should be cautious of it. While failing to develop adequate “analytical, synthetic and holistic thinking” in the process (He and Tao, 2022), they are only slightly mindful of it so far. Second, China still lacks adequate qualified TTT teachers. Only 8.04% were adept in translation technology in the Chinese MTI context in 2018 (Wang et al., 2018). This gap might result in the limited influence exerted by teachers on their students. Another probable explanation is the cognitive tensions between student translators and computers (O’Brien, 2012). Most MTI students (including those in China) do not regard themselves as technology savvy, and they may bear some resistance against those computer programs seeming to be far detached from their traditional translation and language learning (Krüger, 2021). Thus, they may still feel inhibited when learning and using translation technology. In general, these findings add to the existing literature that elucidates students’ low appreciation for translation technology (Man et al., 2020) and that call for cultivating proactive attitudes towards it (Gaspari et al., 2015). Therefore, more attention should be paid to its antecedents, or rather the individual motivational factors underlying students’ ATT. That is why RQ2 was established.

RQ2 explores the structural relations between two individual motivational factors and ATT. As shown in Figure 3, regarding the relationship between TM and ATT, the results indicate that growth translation mindsets positively associate with students’ attitudes towards the effectiveness of translation technology, teacher influence, exhibition to translation technology, and mindfulness about translation technology. These findings complement the extant literature that espouses the facilitating role of growth mindsets in developing positive beliefs about technology-mediated learning (Zarrinabadi et al., 2022). The potential explanations are as follows. First, students with growth translation mindsets believe their translation competence is malleable and could be enhanced through hard work. Such people may give more value to translation technology and probably learn and use it well as they think that their well-intended efforts in this regard would improve their translation. Also, more knowledge and experience could lead to more useful perceptions of translation technology (Yang and Wang, 2019). Second, as proved by past studies, growth mindsets positively predict students’ learning motivation (Zarrinabadi et al., 2022), engagement with teachers (Blackwell et al., 2007), and self-efficacy (Bai and Guo, 2021). Thus, with stronger motivation in learning translation technology and utilizing it for improving translation, students who endorse growth translation mindsets are more likely to go beyond studying the mere technical procedures and become critical and mindful of it. Understandably, in the whole process, they would be more influenced by their teachers. In other words, teachers’ encouragement and enthusiasm for TTT also play a significant role in defining their ATT. Furthermore, given the positive correlations between growth mindsets and self-efficacy (Bai and Guo, 2021), it seems reasonable that students with growth translation mindsets tend to be more confident and resilient and feel less anxious about learning translation technology. Notably, the path coefficients of growth translation mindsets are more significant than those of others, implying that growth translation mindsets are the strongest predictor for all ATT components. Therefore, from the motivational perspective, it can be concluded that growth translation mindsets can operate well to encourage students to develop more positive attitudes towards translation technology.

In addition, the study results also reveal that fixed translation mindsets negatively predict teacher influence on students’ ATT. The possible reason is that students who believe their translation competence is unchangeable might not be easily affected by others, including teachers. However, contrary to our expected hypotheses, the results do not show any significant link between fixed translation mindsets and students’ attitudes towards the effectiveness of translation technology, exhibition to translation technology, and mindfulness about translation technology. This is somewhat surprising because these findings contradict the core assumptions of the mindset theory and run counter to the existing study that has confirmed the negative role of fixed mindsets in students’ attitudes towards technology-mediated learning (Zarrinabadi et al., 2022). Such inconsistency might be explained like this: students with fixed translation mindsets may not like to do translation by themselves as they believe that their translation competence cannot be changed through it. In this sense, such individuals may find some value in translation technology as it can help them to produce fast translations or even fully replace their human work. Correspondingly, for such students, translation technology’s energy-saving and fast production properties might also lead to less defensive and more mindful perceptions. Hence, the unsupported hypotheses imply a kind of fake positive ATT potentially existing among students with fixed translation mindsets. More efforts should be rendered for careful distinction.

As for the relations between FWS and ATT, the results show that future work self-salience positively influences students’ attitudes towards the effectiveness of translation technology and mindfulness about translation technology, while future work self-elaboration only positively predicts students’ exhibition to translation technology. This is probably because that students with a salient representation of their future work aspirations are better conscious of the substantial relevance of translation technology in the translation workplace. Increasing awareness in this respect would lead to more effective perceptions. This also indicates that such individuals are more willing to embrace translation technology and regard learning as a significant part of future career preparation. In such a sense, they would have higher self-awareness to be a mindful and critical TT user. These findings generally echo several studies that advocate future work self as an essential psychological strength in students’ technological learning (Tucker, 2014; Ardies et al., 2015; Taghizadeh and Hajhosseini, 2021). Nevertheless, the correlations between future work self-salience, students’ teacher influence, and exhibition to translation technology are weak. There are two possible explanations for these results. First, future work self-salience represents an aspect of self-concept, motivating proactive future-oriented behaviors mainly through individuals’ self-recognition of discrepancies between the current and the future (Strauss et al., 2012; Taber and Blankemeyer, 2015). This strong self-concept might hardly evoke teacher influence. Meanwhile, future work self-salience denotes the clarity of one’s future aspiration, suggesting that only clear future representation cannot alleviate students’ anxiety in learning and using translation technology. That is to say, in terms of enhancing students’ exhibition to translation technology, only knowing about its importance for a future career may not be adequate, and concrete planning or actions might also be needed. The only supported hypothesized link between future work self-elaboration and students’ exhibition to translation technology could serve as another evidence to interpret this viewpoint. Overall, from the motivational standpoint, future work self-salience and elaboration, both crucial predictors for different ATT components, can stimulate students’ positive attitudes towards translation technology in a collective and complimentary manner.

To sum up, the findings and discussions reported in the study unveil the considerable facilitating effect of growth translation mindsets and indicate the urgent need for relevant pedagogical thinking and research in the TTT context. Moreover, attention should be paid to distinguishing the fake positive ATT potentially brought by fixed translation mindsets. Efforts should also be made to activate both future work self-salience and future work self-elaboration to fully play the antecedent role of future work self in developing students’ positive ATT.

## 7. Implications

The current study examines Chinese MTI students’ ATT and two motivational antecedents in relation to it. It has important implications for TTT in both theoretical and pedagogical ways.

Theoretically, the findings help to reveal two new motivational antecedents that could shape students’ ATT. Specifically, we have uncovered the theoretical value of translation mindsets and future work self as motivational resources for positive attitudes towards translation technology. By integrating them into the ATT model, this study offers a detailed account of how and why growth translation mindsets, fixed translation mindsets, future work self-salience, and future work self-elaboration associate with the four ATT components. Such student-centered research could present a novel perspective in the TTT domain, which primarily focuses on the course design itself: individual factors play a vital role in students’ learning and adoption of translation technology. All these might offer some theoretical implications for future TTT studies.

Pedagogically, the empirical findings have crucial implications for teaching translation technology in the following respects. In the Chinese MTI context, students’ overall attitudes towards translation technology are slightly positive. In such a sense, it is significant to help them to develop positive attitudes.

First, given the dominant effect of growth translation mindsets on all ATT components, changing students’ fixed translation mindsets and encouraging growth mindsets could be an efficient way to improve their ATT. Teachers could employ growth mindsets interventions to promote students’ growth translation mindsets and, consequently, their attitudes towards translation technology. For instance, in their teaching of translation or translation technology, teachers could follow the mastery-oriented approach, emphasizing efforts rather than ability, and stressing process instead of outcomes (Sato and Csizer, 2021). To this end, teachers could use self-referenced assessment (Ames, 1992), namely, evaluating students’ progress with reference to their own development, and avoid unnecessary comparison. In addition, teachers could guide students to set attainable and specific developmental goals. Fulfilling such goals could allow students to feel a sense of agency (Zarrinabadi et al., 2022). Teachers could give more praise and feedback to the students and encourage them to elaborate on their thoughts, for instance, through reflective essays (Cheung, 2019). Besides these, there are multiple other pedagogical strategies that teachers could further explore.

Second, given the weak correlations observed between FMT and AETT, ETT, and MTT, teachers must be mindful of the kind of fake positive attitudes that potentially exist among students with fixed translation mindsets. The unsupported hypotheses suggest that these students might appreciate the fast-production or human-work-replacing value of translation technology as they are reluctant to translate by themselves. Thus, there is a need to raise students’ awareness that human beings are always the center of translation production, and translation technology is meant to assist rather than replace human translation (Vieira, 2019; Rico and Gonzalez Pastor, 2022). In doing so, when teaching translation technology, teachers should focus on the paradigm of machine-assisted human translation instead of human-assisted machine translation.

Furthermore, as future work self is confirmed to influence students’ attitudes towards translation technology directly, it is also necessary to cultivate a salient and elaborate future work self for them. Specifically, future work self-salience could be enhanced through future-oriented initiatives: specialized translation career counseling and career development programs could be made available to students (Lu, 2020). Teachers could create a supportive workplace atmosphere for translation technology learning. More importantly, teachers should encourage students to think about their future translation careers, pay attention to the discrepancy between their current and future self, and strengthen their awareness and control of future career development. Future work self-elaboration could be strengthened through specific future-oriented guidance: efforts could be made to guide students to sort out future career goals and learn about corresponding planning. There is also a need to give students a sense of breaking down their career goals and relating them to their everyday life and study activities (Ling et al., 2022). Additionally, both future work self-salience and elaboration can be upgraded through the career-oriented TTT approach, such as simulating real-work scenarios (Alcina et al., 2007), engaging students in authentic translation projects (He and Tao, 2022), considering how professionals perform (Song and Cheung, 2019; Ma and Cheung, 2020; Wu et al., 2021), enhancing ties with the industry, and integrating translation technology into other core translation courses (Bowker and Marshman, 2010). All of these could bring students closer to the real professional environment and allow them to know about the latest application of translation technology in the workplace. Consequently, all these could help students to be salient and elaborate about their future careers and, in turn, more willing to learn and use translation technology.

## 8. Conclusion

Despite the growing interest in TTT research, there is still little scholarship on students’ attitudes towards translation technology. Through a questionnaire-based survey, this study intended to fill this gap by describing students’ overall attitudes towards translation technology in the Chinese MTI context and exploring how translation mindsets and future work self are related to it. There are several significant findings.

First, the results indicate that students’ overall attitudes towards translation technology are slightly positive in the Chinese MTI context. So far, they perceive translation technology to be slightly effective for translation and are slightly mindful of it. They are slightly influenced by teachers and still feel inhibited when learning and using it. Besides, the results also reveal that growth translation mindsets positively associate with students’ attitudes towards the effectiveness of translation technology, teacher influence, exhibition to translation technology, and mindfulness about translation technology, while fixed translation mindsets only negatively predict students’ teacher influence. Likewise, future work self-salience positively influences students’ attitudes towards the effectiveness of translation technology and mindfulness about translation technology, while future work self-elaboration positively predicts students’ exhibition to translation technology. Among them, growth translation mindsets are the strongest predictor for all ATT components. Taken together, this study has validated the role of translation mindsets and future work self in students’ attitudes towards translation technology. This could provide theoretically meaningful evidence for the student-centered line of research in the TTT domain. These findings could also offer practical pedagogical implications for improving students’ ATT: employing growth mindsets interventions to promote students’ growth translation mindsets, being mindful about the kind of fake positive attitudes potentially existing among students with fixed translation mindsets, and cultivating a salient and elaborate future work self for students through career development programs, specific future-oriented guidance and career-oriented TTT approach.

Nevertheless, a few limitations must be noted for future research. Firstly, the study is limited to a small student group within a single context. Only one hundred and eight grade 2021 MTI students from three Chinese universities participated in this research. A larger sample size involving more than one grade of students from diverse contexts may complement the present findings and provide more profound results. Secondly, the study only relied on self-reported data from a questionnaire-based survey. The self-report results in the current research are only exploratory. Measuring ATT and its motivational antecedents through various data sources (like observations, experiments, and interviews) will present a more valid view. Thirdly, since the study used purposive sampling, a non-probability sampling method, generalizations of the findings to the whole population might be a problem. Fourthly, students’ attitudes towards translation technology might change over time, and the study failed to consider this variable. Therefore, a longitudinal and comparative study may be conducted to exploit the impact of changes in time on students’ ATT and the potential variables. Fifthly, this study only surveyed students’ attitudes towards the umbrella term of translation technology. Translation technology has various categories. Future research could focus on its specific sorts. In addition, the study only examined two motivational antecedents in relation to students’ ATT. Other motivational factors potentially predicting ATT deserve more scholarly attention in further studies.

The current research contributes to TTT literature in several ways. This study, which is descriptive and exploratory, represents an initial effort to examine students’ attitudes towards translation technology and its structural relations with two of its motivational antecedents. The results also emphasize the need to survey further the intersections between ATT and the individual motive system. In sum, this study could serve as a baseline for broader investigations of the human factors associated with students’ translation technology learning and use, which is currently under-researched in TTT.

## Data availability statement

The raw data supporting the conclusions of this article will be made available by the authors, without undue reservation.

## Ethics statement

The studies involving human participants were reviewed and approved by Ethics Committee of School of Foreign Languages of Central South university, Ningxia University and Hainan university. The participants provided the written informed consent to participate in this study.

## Author contributions

ST is responsible for designing the research experiment, analyzing the dataset, and writing the thesis. ZZ and LJ are responsible for collecting the data. All authors contributed to the article and approved the submitted version.

## Funding

This research was supported by the National Social Science Foundation of China (21CYY004).

## Conflict of interest

The authors declare that the research was conducted in the absence of any commercial or financial relationships that could be construed as a potential conflict of interest.

## Publisher’s note

All claims expressed in this article are solely those of the authors and do not necessarily represent those of their affiliated organizations, or those of the publisher, the editors and the reviewers. Any product that may be evaluated in this article, or claim that may be made by its manufacturer, is not guaranteed or endorsed by the publisher.

## References

[ref1] Ajzen I. (1991). The theory of planned behavior. Organ. Behav. Hum. Decis. Process. 50, 179–211. doi: 10.1016/0749-5978(91)90020-T

[ref2] Alcina Caudet A. (2002). Strategies and resources in the teaching of IT applied to translation. Simposi sobre l'Ensenyament a distància i semipresencial de la Tradumàtica, 1–9.

[ref3] Alcina A. Soler V. Granell J. (2007). Translation technology skills acquisition. Perspectives 15, 230–244. doi: 10.1080/13670050802280179

[ref200] Ames C. (1992). Classrooms: Goals, structures, and student motivation. J. Educ. Psychol. 84, 261–271. doi: 10.1037/0022-0663.84.3.261

[ref4] Anderson J. C. Gerbing D. W. (1988). Structural equation modeling in practice: a review and recommended two-step approach. Psychol. Bull. 103, 411–423. doi: 10.1037/0033-2909.103.3.411

[ref5] Ardies J. De Maeyer S. Gijbels D. (2015). A longitudinal study on boys’ and girls’ career aspirations and interest in technology. Res. Sci. Technol. Educ. 33, 366–386. doi: 10.1080/02635143.2015.1060412

[ref6] Austermühl F. (2013). Future (and not-so-future) trends in the teaching of translation technology. Revista Tradumàtica: tecnologies de la traducció 11, 326–337. doi: 10.5565/rev/tradumatica.46

[ref7] Bagozzi R. P. Yi Y. (1988). On the evaluation of structural equation models. J. Acad. Mark. Sci. 16, 74–94. doi: 10.1177/009207038801600107

[ref8] Bai B. Guo W. (2021). Motivation and self-regulated strategy use: relationships to primary school students’ English writing in Hong Kong. Lang. Teach. Res. 25, 378–399. doi: 10.1177/1362168819859921

[ref9] Biau-Gil J. R. Pym A. (2006). “Technology and translation (a pedagogical overview)” in Translation technology and its teaching. eds. Pym A. Perestrenko A. Starink B. (Tarragona: Intercultural Studies Group, Universitat Rovira i Virgili), 5–19.

[ref10] Blackwell L. S. Trzesniewski K. H. Dweck C. S. (2007). Implicit theories of intelligence predict achievement across an adolescent transition: a longitudinal study and an intervention. Child Dev. 78, 246–263. doi: 10.1111/j.1467-8624.2007.00995.x, PMID: 17328703

[ref11] Blazar D. (2018). Validating teacher effects on students’ attitudes and behaviors: evidence from random assignment of teachers to students. Educ. Finance and Policy 13, 281–309. doi: 10.1162/edfp_a_00251

[ref12] Blazar D. Kraft M. A. (2017). Teacher and teaching effects on students’ attitudes and behaviors. Educ. Eval. Policy Anal. 39, 146–170. doi: 10.3102/0162373716670260, PMID: 28931959 PMC5602565

[ref13] Bowker L. Marshman E. (2010). Toward a model of active and situated learning in the teaching of computer-aided translation: introducing the CERTT project. J. Translation Stud. 13, 199–226.

[ref14] Briggs N. (2018). Neural machine translation tools in the language learning classroom: Students' use, perceptions and analyses. JALT CALL J. 14, 2–24. doi: 10.29140/jaltcall.v14n1.221

[ref15] Byrne B. M. (2013). Structural equation modeling with AMOS: Basic concepts, applications, and programming. New York: Routledge.

[ref16] Calvo E. (2017). Servicios de valor añadido en contextos situacionales en traducción: de los proyectos al portafolio. Revista Digital de Investigación en Docencia Universitaria 11, 136–154. doi: 10.19083/ridu.11.576

[ref17] Cheung A. K. F. (2019). “The hidden curriculum revealed in study trip reflective essays” in The evolving curriculum in interpreter and translator education: Stakeholder perspectives and voices. eds. David B. Austermühl F. Raído V. E. (Netherlands: John Benjamins), 393–408.

[ref18] Cheung A. K. F. (2022). COVID-19 and interpreting. INContext: Stud. Translation Intercultura. 2, 9–13. doi: 10.54754/incontext.v2i2.26

[ref19] Coetzee M. (2019). “The value of future-fit psychosocial career self-management capabilities in sustaining career wellbeing” in Theory,research and dynamics of career wellbeing: Becoming fit for the future, eds. I. Potgieter, N. Ferreira and M. Coetzee (Springer, Cham), 139–157.

[ref20] Cronin M. (2010). The translation crowd. Revista Tradumàtica: tecnologies de la traducció 8, 1–7. doi: 10.5565/rev/tradumatica.100

[ref21] Doherty S. Kenny D. (2014). The design and evaluation of a statistical machine translation syllabus for translation students. Interpreter and Translator Trainer 8, 295–315. doi: 10.1080/1750399X.2014.937571

[ref22] Doherty M. Mitchell E. A. O'Neill S. (2011). Attitudes of healthcare workers towards older people in a rural population: a survey using the Kogan scale. Nurs. Res. Pract. 2011, 1–7. doi: 10.1155/2011/352627, PMID: 21994823 PMC3170018

[ref23] Doherty S. Moorkens J. (2013). Investigating the experience of translation technology labs: pedagogical implications. J. Specialised Translation 19, 122–136.

[ref24] Dweck C. S. (2006). Mindset: The new psychology of success. New York: Random House.

[ref25] Dweck C. S. Leggett E. L. (1988). A social-cognitive approach to motivation and personality. Psychol. Rev. 95, 256–273. doi: 10.1037/0033-295X.95.2.256

[ref26] Ehrensberger-Dow M. O’Brien S. (2015). Ergonomics of the translation workplace: potential for cognitive friction. Translation Spaces 4, 98–118. doi: 10.1075/ts.4.05ehr

[ref27] EMT. (2017). EMT competence framework 2017. Available online at: https://ec.europa.eu/info/sites/info/files/emt_competence_fwk_2017_en_web.pdf

[ref28] Enríquez Raído V. (2013). Teaching translation technologies “everyware”: towards a self-discovery and lifelong learning approach. Tradumàtica 11, 275–285. doi: 10.5565/rev/tradumatica.52

[ref29] Esteban-Millat I. Martínez-López F. J. Pujol-Jover M. Gázquez-Abad J. C. Alegret A. (2018). An extension of the technology acceptance model for online learning environments. Interact. Learn. Environ. 26, 895–910. doi: 10.1080/10494820.2017.1421560

[ref30] Fornell C. Larcker D. F. (1981). Evaluating structural equation models with unobservable variables and measurement error. J. Mark. Res. 18, 39–50. doi: 10.1177/002224378101800104

[ref31] Gaspari F. Almaghout H. Doherty S. (2015). A survey of machine translation competences: insights for translation technology educators and practitioners. Perspectives 23, 333–358. doi: 10.1080/0907676X.2014.979842

[ref32] González Davies M. Enríquez Raído V. (2016). Situated learning in translator and interpreter training: bridging research and good practice. Interpreter and Translator Trainer 10, 1–11. doi: 10.1080/1750399X.2016.1154339 PMC4959122 27499805

[ref33] González Pastor D. Rico C. (2021). POSEDITrad: La traducción automática y la posedición para la formación de traductores e intérpretes. Revista Digital De Investigación En Docencia Universitaria 15:1213. doi: 10.19083/10.19083/ridu.2021.1213

[ref34] Guan Y. J. Guo Y. Bond M. H. Cai Z. J. Zhou X. Xu J. W.. (2014). New job market entrants’ future work self, career adaptability and job search outcomes: examining mediating and moderating models. J. Vocat. Behav. 85, 136–145. doi: 10.1016/j.jvb2014.05.003

[ref35] Guerberof Arenas A. Moorkens J. (2019). Machine translation and post-editing training as part of a master’s programme. J. Specialised Translation 31, 217–238.

[ref36] Hair J. F. Black B. Babin B. Anderson R. E. Tatham R. L.. (2006). *Multivariate Data Analysi*s. 6th. London: Pearson.

[ref37] Hayduk L. A. (1987). Structural equation modeling with LISREL: Essentials and advances. United States: Jhu Press.

[ref38] He S. Hao Y. Liu S. Liu H. Li H. (2022). Research on translation technology teaching in Chinese publications and in international English-language publications (1999-2020): a bibliometric analysis. Interpreter and Translator Trainer 16, 275–293. doi: 10.1080/1750399X.2022.2101848

[ref39] He Y. Tao Y. (2022). Unity of knowing and acting: an empirical study on a curriculum approach to developing students’ translation technological thinking competence. Interpreter and Translator Trainer 16, 348–366. doi: 10.1080/1750399X.2022.2101849

[ref40] Hoyle R. H. Sherrill M. R. (2006). Future orientation in the self-system: possible selves, self-regulation, and behavior. J. Pers. 74, 1673–1696. doi: 10.1111/j.1467-6494.2006.00424.x, PMID: 17083662

[ref41] Husman J. Lens W. (1999). The role of the future in student motivation. Educ. Psychol. 34, 113–125. doi: 10.1207/s15326985ep3402_4

[ref42] Jiménez-Crespo M. (2013). Translation and web localization. London: Routledge.

[ref43] Jiménez-Crespo M. Tercedor M. (2011). Applying corpus data to define needs in web localization training. Meta: J. des traducteurs/Meta: Translators’ J. 56, 998–1021. doi: 10.7202/1011264ar

[ref44] Kenny D. Doherty S. (2014). Statistical machine translation in the translation curriculum: overcoming obstacles and empowering translators. Interpreter and Translator Trainer 8, 276–294. doi: 10.1080/1750399X.2014.936112

[ref45] Khajavy G. H. MacIntyre P. D. Hariri J. (2021). A closer look at grit and language mindset as predictors of foreign language achievement. Stud. Second. Lang. Acquis. 43, 379–402. doi: 10.1017/S0272263120000480

[ref46] Killman J. (2018). “A context-based approach to introducing translation memory in translator training” in Translation, globalisation and translocation: The classroom and beyond. ed. Concepción G. (London: Palgrave Macmillan), 137–159.

[ref47] Kim H. Y. (2013). Statistical notes for clinical researchers: assessing normal distribution (2) using skewness and kurtosis. Restorative Dentistry & Endodontics 38, 52–54. doi: 10.5395/rde.2013.38.1.52, PMID: 23495371 PMC3591587

[ref48] Kline R. B. (2005). Principles and practice of structural equation modeling (2nd). New York: The Guilford Press.

[ref49] Kline R. B. (2011). Principles and practice of structural equation modeling (3rd). New York: The Guillford Press.

[ref400] Kline R. B. (2015). Principles and practice of structural equation modeling. (3rd Edn.). New York: The Guillford Press.

[ref50] Krüger R. (2016). Situated LSP translation from a cognitive translational perspective. Lebende Sprachen 61, 297–332. doi: 10.1515/les-2016-0014

[ref51] Krüger R. (2018). Technologieinduzierte Verschiebungen in der Tektonik der Translationskompetenz. Transfusion 11, 104–137.

[ref52] Krüger R. (2021). An online repository of python resources for teaching machine translation to translation students. Current Trends in Translation Teach. Learn. 8, 4–30. doi: 10.51287/cttle20212

[ref53] Lai Y. Saab N. Admiraal W. (2022). University students’ use of mobile technology in self-directed language learning: using the integrative model of behavior prediction. Comput. Educ. 179:104413. doi: 10.1016/j.compedu.2021.104413

[ref54] Liaw S. S. (2002). An internet survey for perceptions of computer and world wide web: relationship, prediction, and difference. Comput. Hum. Behav. 18, 17–35. doi: 10.1016/S0747-5632(01)00032-2

[ref55] Lin K. M. (2011). E-learning continuance intention: moderating effects of user e-learning experience. Comput. Educ. 56, 515–526. doi: 10.1016/j.compedu.2010.09.017

[ref56] Ling H. Teng S. Liu X. Wu J. Gu X. (2022). Future work self salience and future time perspective as serial mediators between proactive personality and career adaptability. Front. Psychol. 13:824198. doi: 10.3389/fpsyg.2022.82419, PMID: 35572329 PMC9094421

[ref57] Liu K. Kwok H. L. Liu J. Cheung A. K. F. (2022). Sustainability and influence of machine translation: perceptions and attitudes of translation instructors and learners in Hong Kong. Sustain. For. 14:6399. doi: 10.3390/su14116399

[ref58] Lou N. M. Noels K. A. (2017). Measuring language mindsets and modeling their relations with goal orientations and emotional and behavioral responses in failure situations. Mod. Lang. J. 101, 214–243. doi: 10.1111/modl.12380

[ref59] Lou N. M. Noels K. A. (2020). Breaking the vicious cycle of language anxiety: growth language mindsets improve lower-competence ESL students’ intercultural interactions. Contemp. Educ. Psychol. 61:101847. doi: 10.1016/j.cedpsych.2020.101847

[ref60] Lu W. C. (2020). Future work-self salience and proactive career behavior among college student-athletes in Taiwan: a career construction model of adaptation. J. Hosp. Leis. Sport Tour. Educ. 27:100259. doi: 10.1016/j.jhlste.2020.100259

[ref61] Ma X. Cheung A. K. F. (2020). Language interference in English-Chinese simultaneous interpreting with and without text. Babel 66, 434–456. doi: 10.1075/babel.00168.che

[ref62] Man D. Mo A. Chau M. H. O’Toole J. M. Lee C. (2020). Translation technology adoption: evidence from a postgraduate programme for student translators in China. Perspectives 28, 253–270. doi: 10.1080/0907676X.2019.1677730

[ref63] Martínez S. M. Benítez P. F. (2009). Terminological competence in translation. Terminology. Int. J. Theoretical App. Issues in Specialized Commun. 15, 88–104. doi: 10.1075/term.15.1.05mon

[ref64] Massey G. Ehrensberger-Dow M. (2017). Machine learning: implications for translator education. Lebende Sprachen 62, 300–312. doi: 10.13140/RG.2.2.12968.98562

[ref65] Mellinger C. D. (2018). Problem-based learning in computer-assisted translation pedagogy. HERMES-J. Lang. Commun. Business 57, 195–208. doi: 10.7146/hjlcb.v0i57.106205

[ref66] Mercer S. Ryan S. (2010). A mindset for EFL: learners’ beliefs about the role of natural talent. ELT J. 64, 436–444. doi: 10.1093/elt/ccp083

[ref67] Mitchell-Schuitevoerder R. (2020). A project-based approach to translation technology. London: Routledge.

[ref300] Mo A. Man D. (2017). The ecosystem of translator workstation: Learning electronic tools in a training program for professional translators in China. Babel 63, 401–422. doi: 10.1075/babel.63.3.06aip

[ref68] Moorkens J. (2018). What to expect from neural machine translation: a practical in-class translation evaluation exercise. Interpreter and Translator Trainer 12, 375–387. doi: 10.1080/1750399X.2018.1501639

[ref69] Ngampornchai A. Adams J. (2016). Students’ acceptance and readiness for E-learning in northeastern Thailand. Int. J. Educ. Technol. High. Educ. 13, 1–13. doi: 10.1186/s41239-016-0034-x

[ref70] Nunes Vieira L. Zhang X. Yu G. (2021). “Click next”: on the merits of more student autonomy and less direct instruction in CAT teaching. Interpreter and Translator Trainer 15, 411–429. doi: 10.1080/1750399X.2021.1891515

[ref71] O’Brien S. (2012). Translation as human-computer interaction. Translation spaces 1, 101–122. doi: 10.1075/ts.1.05obr

[ref72] PACTE (2018). Competence levels in translation: working towards a European framework. Interpreter and Translator Trainer 12, 111–131. doi: 10.1080/1750399x.2018.1466093

[ref73] Papi M. Rios A. Pelt H. Ozdemir E. (2019). Feedback-seeking behavior in language learning: basic components and motivational antecedents. Mod. Lang. J. 103, 205–226. doi: 10.1111/modl.12538

[ref74] Papi M. Wolff D. Nakatsukasa K. Bellwoar E. (2021). Motivational factors underlying learner preferences for corrective feedback: language mindsets and achievement goals. Lang. Teach. Res. 25, 858–877. doi: 10.1177/13621688211018808

[ref75] Pym A. (2011). What technology does to translating. Translation & Interpreting 3, 1–9. doi: 10.12807/t&i.v3i1.121

[ref76] Pym A. Perekrestenko A. Starink B. (2006). Translation technology and its teaching. Tarragona: Universitat Rovira i Virgili (Intercultural Studies Group).

[ref77] Rahimi M. Zhang L. J. (2022). Effects of an engaging process-genre approach on student engagement and writing achievements. Read. Writ. Q. 38, 487–503. doi: 10.1080/10573569.2021.1982431

[ref78] Rico C. (2017). The ePortfolio: constructing learning in translation technology. Interpreter and Translator Trainer 11, 79–95. doi: 10.1080/1750399x.2017.1306995

[ref79] Rico C. Gonzalez Pastor D. (2022). The role of machine translation in translation education: a thematic analysis of translator educators' beliefs. Translation & Interpreting 14, 177–197. doi: 10.12807/ti.114201.2022.a010

[ref80] Rodríguez-Castro M. (2018). An integrated curricular design for computer-assisted translation tools: developing technical expertise. Interpreter and Translator Trainer 12, 355–374. doi: 10.1080/1750399X.2018.1502007

[ref81] Rodríguez-Inés P. (2013). Electronic target-language specialised corpora in translator education. Babel 59, 57–75. doi: 10.1075/babel.59.1.04rod

[ref82] Rothwell A. Svoboda T. (2019). Tracking translator training in tools and technologies: findings of the EMT survey 2017. J. Specialised Translation 32, 26–60.

[ref83] Sánchez Ramos M. D. M. (2022). Public service interpreting and translation training: a path towards digital adaptation to machine translation and post-editing. Interpreter and Translator Trainer 16, 294–308. doi: 10.1080/1750399X.2022.2092829

[ref84] Sato M. Csizer K. (2021). Introduction: combining learner psychology and ISLA research: intersections in the classroom. Lang. Teach. Res. 25, 839–855. doi: 10.1177/13621688211044237

[ref85] Sha L. Wang X. Ma S. Mortimer T. A. (2022). Investigating the effectiveness of anonymous online peer feedback in translation technology teaching. Interpreter and Translator Trainer 16, 325–347. doi: 10.1080/1750399X.2022.2097984

[ref86] Shuttleworth M. (2017). Cutting teeth on translation technology: how students at University College London are being trained to become tomorrow’s translators. Tradução em Revista 2017, 18–38. doi: 10.17771/PUCRio.TradRev.30595

[ref87] Sikora I. (2014). The need for CAT training within translator training programmes. TRAlinea Special Issue: Challenges in Translation Pedagogy, 1–6.

[ref88] Song S. Cheung A. K. F. (2019). Disfluency in relay and non-relay simultaneous interpreting: an initial exploration. Forum 17, 1–19. doi: 10.1075/forum.18016.che

[ref89] Strauss K. Griffin M. A. Parker S. K. (2012). Future work selves: how salient hoped-for identities motivate proactive career behaviors. J. Appl. Psychol. 97, 580–598. doi: 10.1037/a0026423, PMID: 22122111

[ref90] Tabachnick B. G. Fidell L. S. (2018). Using multivariate statistics (7th). London: Pearson.

[ref91] Taber B. J. Blankemeyer M. (2015). Future work self and career adaptability in the prediction of proactive career behaviors. J. Vocat. Behav. 86, 20–27. doi: 10.1016/j.jvb.2014.10.005

[ref92] Taghizadeh M. Hajhosseini F. (2021). Investigating a blended learning environment: contribution of attitude, interaction, and quality of teaching to satisfaction of graduate students of TEFL. Asia Pac. Educ. Res. 30, 459–469. doi: 10.1007/s40299-020-00531-z

[ref93] Taherdoost H. (2019). What is the best response scale for survey and questionnaire design; review of different lengths of rating scale/attitude scale/Likert scale. Int. J. Academic Res. Manag. 8, 1–10.

[ref500] Tao Y. (2019). “Problems and Solutions: The Undergraduate Translator Education in Chinese Mainland,” in Restructuring Translation Education: Implications from China for the Rest of the World. eds. F. Yue, Y. Tao, H. Wang, Q. Cui and B. Xu (Singapore: Springer), 29–40.

[ref94] Tao Y. Wang H. (2022). Introduction to the special issue translation technology teaching: views and visions. Interpreter and Translator Trainer 16, 271–274. doi: 10.1080/1750399X.2022.2101851

[ref95] Tao Y. Xie M. Zhou Q. Li X. Cheng S.. (2020). Becoming a technical writer. Shanghai: Fudan University Press.

[ref96] Tucker S. Y. (2014). Transforming pedagogies: integrating 21st century skills and web 2.0 technology. Turkish online journal of. Distance Educ. 15, 166–173. doi: 10.17718/tojde.32300

[ref97] Vandewaetere M. Desmet P. (2009). Introducing psychometrical validation of questionnaires in CALL research: the case of measuring attitude towards CALL. Comput. Assist. Lang. Learn. 22, 349–380. doi: 10.1080/09588220903186547

[ref98] Vargas-Sierra C. (2014). “Innovación didáctica en traducción especializada: sobre la enseñanza virtual de traducción de páginas web de contenido económico” in Traducción económica: entre profesión, formación y recursos documentales. ed. GallegoHernández D. (Soria: Vertere), 110–130.

[ref99] Varlotta L. (2018). Designing a model for the new Liberal arts. Lib. Educ. 104, 44–51.

[ref100] Vieira L. N. (2019). “Post-editing of machine translation” in The Routledge handbook of translation and technology. ed. O’Hagan M. (London: Routledge), 319–313.

[ref101] Waller L. Papi M. (2017). Motivation and feedback: how implicit theories of intelligence predict L2 writers’ motivation and feedback orientation. J. Second. Lang. Writ. 35, 54–65. doi: 10.1016/j.jslw.2017.01.004

[ref102] Wang H. Li D. Li L. (2018). Fanyi zhuanye shuoshi (MTI) fanyi jishu jiaoxue yanjiu [translation technology teaching in MTI programs in China:problems and suggestions]. Waiyu dianhua jiaoxue 3, 76–82.

[ref103] Wang L. Wang X. (2021). Building virtual communities of practice in post-editing training: a mixed-method quasi-experimental study. J. Specialised Translation 36, 193–219.

[ref104] Wenden A. L. (1991). Learner strategies for learner autonomy. London: Prentice Hall.

[ref105] Wu B. Cheung A. K. F. Xing J. (2021). Learning Chinese political formulaic phraseology from a self-built bilingual United Nations security council corpus: a pilot study. Babel 67, 500–521. doi: 10.1075/babel.00233.wu

[ref106] Yan D. Wang J. (2022). Teaching data science to undergraduate translation trainees: pilot evaluation of a task-based course. Front. Psychol. 13:939689. doi: 10.3389/fpsyg.2022.939689, PMID: 35992492 PMC9381704

[ref107] Yang Y. Wang X. (2019). Modeling the intention to use machine translation for student translators: an extension of technology acceptance model. Comput. Educ. 133, 116–126. doi: 10.1016/j.compedu.2019.01.015

[ref108] You C. J. Dornyei Z. (2016). Language learning motivation in China: results of a large-scale stratified survey. Appl. Linguis. 37, 495–519. doi: 10.1093/applin/amu046

[ref109] Zarrinabadi N. Rezazadeh M. Mohammadzadeh Mohammadabadi A. (2022). L2 grit and language mindsets as predictors of EFL learners’ attitudes toward effectiveness and value of CALL. Comput. Assist. Lang. Learn. 1-18, 1–18. doi: 10.1080/09588221.2022.2108061

[ref110] Zhang X. Vieira L. N. (2021). CAT teaching practices: an international survey. J. Specialised Translation 36, 99–124.

[ref111] Zhang M. Ye M. L. Peng J. Chen Y. S. (2016). Future work self: concept, measurement and related research. Adv. Psychol. Sci. 24, 794–803. doi: 10.3724/SP.J.1042.2016.00794

